# 25-Hydroxycholesterol Restricts Japanese Encephalitis Virus via Metabolic Suppression of the SREBP2-Mediated Signaling Axis

**DOI:** 10.3390/microorganisms14040740

**Published:** 2026-03-26

**Authors:** Xinlei Liu, Yu Gu, Yuanyuan Yang, Xinran Li, Yu Dai, Ruiqin Zhang, Jiahui Li, Haodong Chen, Yi Zheng, Rui Wu

**Affiliations:** 1 Research Center for Swine Diseases, College of Veterinary Medicine, Sichuan Agricultural University, Chengdu 611130, China; leslieliu1998@163.com (X.L.); gu15198666942@163.com (Y.G.); 15111958566@163.com (Y.Y.); lxinran2026@163.com (X.L.); daiyu0102@163.com (Y.D.); zhangruiqin2026@163.com (R.Z.); 13002311849@163.com (J.L.); 18113013960@163.com (H.C.); zhengyi132@126.com (Y.Z.); 2 Sichuan Science-Observation Experiment Station of Veterinary Drugs and Veterinary Diagnostic Technology, Ministry of Agriculture, Chengdu 611330, China; 3 National Animal Experiments Teaching Demonstration Center, Sichuan Agricultural University, Chengdu 611330, China

**Keywords:** SREBP2, 25HC, HMGCR, cholesterol biosynthesis, Japanese encephalitis virus

## Abstract

Host lipid metabolism is a critical determinant of viral pathogenesis. Although the interferon-inducible cholesterol 25-hydroxylase (CH25H) typically acts as a broad-spectrum antiviral protein, its expression and regulatory patterns during Japanese Encephalitis Virus (JEV) infection display unique features. Here, we demonstrate that 25-hydroxycholesterol (25HC), the product of CH25H, potently inhibits JEV proliferation by suppressing SREBP2 activation. Distinct from the majority of viral infections that induce CH25H upregulation, JEV infection elicits a transient reduction in CH25H abundance immediately after infection, coupled with a persistent elevation in SREBP2 expression. This inverse correlation suggests that JEV actively suppresses the CH25H-mediated metabolic checkpoint to maintain a cholesterol-synthetic environment favorable for replication. By pharmacologically simulating the activity of 25HC, we further verify that targeting the SREBP2 signaling axis can efficiently counteract this virally induced metabolic reprogramming. Our study identifies CH25H downregulation and concomitant SREBP2 activation as a key metabolic signature of JEV pathogenesis.

## 1. Introduction

As a neurotropic flavivirus, Japanese encephalitis virus (JEV) persists as a leading causative agent of viral encephalitis throughout East Asia and the Western Pacific region, accounting for an estimated 30,000–50,000 clinical cases and nearly 10,000 fatal outcomes every year [1,2]. Maintained in an enzootic cycle involving Culex mosquitoes, birds, and pigs, JEV poses a significant threat to both public health and the swine industry [3]. Currently, no specific antiviral therapeutics are available, highlighting the critical need to elucidate the molecular basis of JEV pathogenesis. The JEV virion encapsulates a positive-sense RNA genome within an icosahedral nucleocapsid, surrounded by a lipid bilayer embedded with envelope (E) proteins. This lipid envelope serves not only as a structural boundary but also as a critical determinant of infectivity, as the E protein mediates viral attachment and fusion within host endosomes via specific lipid interactions [4,5].

Throughout the replication cycle of flaviviruses, cholesterol-enriched lipid raft microdomains have been well documented to facilitate the cellular entry of dengue virus (DENV) [6] and drive Akt phosphorylation triggered by flavivirus infection [7]. Infection with DENV promotes the intracellular accumulation of cholesterol within viral replication complexes via the activation of 3-hydroxy-3-methylglutaryl-CoA reductase (HMGCR) [8,9]. Likewise, West Nile virus (WNV) reshapes cholesterol homeostasis in host cells by enhancing de novo cholesterol biosynthesis and redirecting cholesterol trafficking to subcellular membranes supporting viral replication [10]. In the case of JEV, the virus delivers its positive-sense RNA genome into host cell cytoplasm through two sequential key steps: receptor-mediated endocytosis, followed by membrane fusion triggered by the acidic microenvironment within endosomes [11]. Following these early infection events, de novo synthesized viral components undergo assembly and encapsidation at the membrane of the host endoplasmic reticulum (ER), before mature virions are released from cells via the exocytic pathway. Host cellular membrane systems serve as critical regulators across multiple stages of the JEV infectious cycle, with an especially indispensable role in the viral entry process. Accumulating evidence from prior studies has confirmed that lipid rafts exert a pivotal regulatory function in JEV entry and genome replication; specifically, disruption of lipid rafts via cholesterol depletion using methyl-β-cyclodextrin (MβCD) was found to significantly attenuate JEV infection in neuronal cells [12].

As a highly abundant lipid component of eukaryotic cellular membranes, cholesterol is indispensable for maintaining membrane compaction, structural integrity, and bilayer fluidity. The replicative life cycle of numerous viruses, encompassing core processes such as cellular entry and progeny virion budding, is tightly modulated by the subcellular distribution of cholesterol within host membranes; consequently, viral propagation is markedly impaired when the de novo cholesterol biosynthesis pathway is blocked [13]. 25-hydroxycholesterol (25HC) is a bioactive oxysterol metabolite generated via site-specific oxidation of the cholesterol backbone [14], an enzymatic reaction specifically catalyzed by cholesterol 25-hydroxylase (CH25H), a functional protein encoded by an interferon-stimulated gene (ISG) [15,16]. Extensive evidence derived from in vitro and in vivo studies has verified that 25HC possesses broad-spectrum antiviral capabilities against multiple enveloped viruses. Representative examples include Kaposi’s sarcoma-associated herpesvirus (KSHV), human immunodeficiency virus type 1 (HIV-1), Ebola virus, West Nile virus (WNV), and Japanese encephalitis virus (JEV) [17,18]. This antiviral function is achieved primarily by regulating intracellular sterol biosynthesis and subcellular trafficking pathways. From a mechanistic perspective, 25HC remodels the spatial organization of cholesterol within the plasma membrane via multiple synergistic pathways: it enhances the membrane accessibility of free cholesterol, reduces total free cholesterol levels in the plasma membrane [19], upregulates intracellular cholesterol esterification, and accelerates cholesterol transport to the endoplasmic reticulum (ER) [20]. On this basis, it is reasonable to hypothesize that 25HC exerts its anti-JEV efficacy by disrupting lipid raft-dependent endocytic processes, which is mediated by the remodeling of cholesterol organization within the host plasma membrane. Notably, Zika virus (ZIKV) infection has been reported to significantly enhance the recruitment of sterol regulatory element-binding protein (SREBP) family transcription factors to the promoter regions of lipogenic genes [21,22]. In both primary and cultured macrophage models, DENV2 infection markedly promotes intracellular lipid droplet biogenesis, as well as the transcriptional upregulation of key genes implicated in the cholesterol synthesis cascade [9,23]. However, the exact molecular targets of 25HC in the setting of JEV infection, particularly its functional impact on the sterol regulatory element-binding protein 2 (SREBP2) pathway—the master transcriptional regulator of mammalian cholesterol biosynthesis—remain largely uncharacterized.

In this study, we identify the SREBP2 signaling axis as a critical determinant of JEV susceptibility. We demonstrate that 25HC exerts its antiviral activity by blocking the proteolytic activation of SREBP2, thereby severing the cholesterol supply required for viral propagation. Conversely, JEV infection triggers a robust upregulation of SREBP2 to sustain lipogenesis, a process further facilitated by the transcriptional downregulation of the upstream inhibitor CH25H. By pharmacologically targeting SREBP2, we recapitulate the antiviral state, establishing the SREBP2 pathway as a pivotal regulator for host–virus metabolic conflict and a promising therapeutic target.

## 2. Materials and Methods

### 2.1. Cell Culture and Virus

The porcine kidney epithelial PK-15 cell line (ATCC catalog No. CCL-33; Manassas, VA, USA) was maintained in Dulbecco’s Modified Eagle’s Medium (DMEM; Gibco, Grand Island, NY, USA). PK-15 is an epithelial cell line isolated from the kidney of an adult pig and has been widely used for the identification and functional verification of host genes implicated in JEV infection [24,25,26,27,28,29]. Complete culture medium was supplemented with 10% fetal bovine serum (FBS; ExCell Bio, Suzhou, China) and a 1% penicillin–streptomycin antibiotic cocktail (Beyotime, Shanghai, China), while the medium used during viral infection was supplemented with 2% FBS and the same concentration of antibiotics. All cell cultures were incubated in a cell incubator at 37 °C with 5% CO_2_. The JEV strain SCYA201201-1 (GenBank accession No. KU508408.1), with a confirmed infectious titer of 5.8 × 10^7^ plaque-forming units (PFU)/mL, was propagated, titrated, and stably preserved in our laboratory.

### 2.2. Antibodies and Reagents

A rabbit polyclonal antibody (pAb) targeting the envelope (E) protein of Japanese encephalitis virus (JEV) was obtained from GeneTex (San Antonio, TX, USA). A rabbit monoclonal antibody (mAb) against β-actin, fluorescein isothiocyanate (FITC)-conjugated goat anti-rabbit IgG, and horseradish peroxidase (HRP)-linked goat anti-rabbit IgG were all sourced from ABclonal (Wuhan, China). A rabbit mAb recognizing SREBP2 (clone F1X2D) was procured from Cell Signaling Technology (CST; Danvers, MA, USA). Rabbit monoclonal antibody against HMGCR (clone YA3518) was acquired from MedChemExpress (MCE; Monmouth Junction, NJ, USA). 25-hydroxycholesterol (25HC, catalog No. T4717), fatostatin (catalog No. T9266), and Cell Counting Kit-8 (CCK-8) assay reagent were all obtained from TargetMol (Shanghai, China). The filipin complex for intracellular cholesterol staining was sourced from MedChemExpress (MCE; Monmouth Junction, NJ, USA).

### 2.3. Real-Time Quantitative PCR (RT-qPCR) Assay

As the major structural protein in Japanese encephalitis virus (JEV) and other flaviviruses, the envelope (E) protein exerts critical functions. It mediates viral cellular entry through its interactions with host receptors and participation in membrane fusion, while also acting as a key target for antibody-mediated neutralization [30,31].

Accordingly, increased expression of the E gene is recognized as a reliable indicator of JEV infection and replication [32].

Total cellular RNA was isolated from cultured cells using the Rapure Total RNA Mini Kit (MaGen, Guangzhou, China). Reverse transcription (RT) was conducted using the PrimeScript™ FAST RT Reagent Kit equipped with gDNA Eraser (Perfect Real Time; Takara Bio Inc., Tokyo, Japan), so as to remove genomic DNA contamination and generate cDNA. Quantitative real-time polymerase chain reaction (qRT-PCR) was performed on the synthesized cDNA using Tli RNaseH Plus (Takara Bio Inc.) with a CFX Connect™ RT-PCR Detection System (Bio-Rad Laboratories, Hercules, CA, USA). The resulting qRT-PCR data were analyzed using CFX Maestro 2.3 (Bio-Rad Laboratories).

Relative mRNA expression levels were calculated employing the 2^−ΔΔCt^ method. β-actin was selected as the internal reference gene for each sample, and the fold change in target gene expression was normalized relative to the mock-infected control group.

All primers utilized for qRT-PCR in the present study are listed in Table 1, and these primers were synthesized by Sangon Biotech (Shanghai, China).

### 2.4. Cytotoxicity Assay

Cell viability was evaluated following the standard protocol of the Cell Counting Kit-8 (CCK-8) assay. Briefly, target cells were plated in 96-well culture plates and exposed to varying concentrations of the test drugs. Following a minimum of 24 h of drug treatment, the culture medium was aspirated, and fresh medium supplemented with CCK-8 reagent was added to each well at a volume of 10 μL per well; the cells were subsequently incubated for an additional 2 h under dark conditions.

The absorbance (A) at a wavelength of 450 nm was detected using a microplate reader. The percentage of viable cells was calculated in accordance with the formula provided by the CCK-8 reagent manufacturer.

### 2.5. Indirect Immunofluorescence (IFA) Assay

PK-15 cells were plated onto 12-well culture plates containing cell climbing slides, followed by infection with JEV at a multiplicity of infection (MOI) of 0.01 and incubation at 37 °C for 36 h. After the incubation period, the cells were rinsed three times with phosphate-buffered saline (PBS), fixed with 4% paraformaldehyde solution for 30 min, and then permeabilized using a mixture of 0.01% Triton-X-100 and 0.5% Saponin for 5 min at room temperature.

Subsequently, the cells were rinsed another three times with PBS, blocked with 2% bovine serum albumin (BSA) at 37 °C for 1 h, and then incubated with rabbit anti-JEV E protein primary antibody (1:250 dilution) at 4 °C overnight. Following the primary antibody incubation, the cells were washed thoroughly with PBST (PBS supplemented with 0.05% Tween-20) and then incubated with FITC-conjugated goat anti-rabbit secondary antibody (1:500 dilution) at 37 °C for 1 h.

After secondary antibody incubation, the cells were rinsed again with PBST and stained with 4′,6-diamidino-2-phenylindole (DAPI; Solarbio, Beijing, China) or propidium iodide (PI; Coolaber, Beijing, China) at 37 °C for 10 min to label cell nuclei. Finally, the stained cells were visualized and imaged using a fluorescence microscope (Olympus BX63, Tokyo, Japan).

### 2.6. Cholesterol Staining

Briefly, PK cells exposed to 25HC or Fatostatin for 6 h were rinsed three times with pre-cooled PBS and fixed in 4% paraformaldehyde solution for 30 min. Subsequently, the cells were rinsed again with pre-cooled PBS and incubated with filipin complex solution at a concentration of 50 μg/mL for 10 min at 37 °C.

Following three additional rinses with PBS to remove excess staining solution, the cells were observed and analyzed using a fluorescence microscope.

### 2.7. Western Blot Analysis

Cells cultured in six-well plates were handled according to the specific protocols of different experimental groups. Following the respective treatments, the cells were rinsed twice with pre-cooled PBS to remove residual culture medium.

Ice-cold RIPA lysis buffer (Beyotime, Shanghai, China) supplemented with 1 mM phenylmethylsulfonyl fluoride (PMSF; Solarbio, Beijing, China) was employed for cell sample lysis, with an incubation period of 5 min on ice. The cell lysates were then boiled for 10 min to denature proteins, followed by electrophoresis using SDS–polyacrylamide gel electrophoresis (SDS–PAGE) to separate proteins by molecular weight.

Subsequently, the separated proteins were transferred onto polyvinylidene difluoride (PVDF) membranes (Vazyme, Nanjing, China). After transfer, the PVDF membranes were blocked with 5% non-fat milk dissolved in TBST at room temperature for 1 h, and then incubated with the corresponding primary antibodies at 4 °C overnight.

Following three rounds of washing with TBST (Tris-buffered saline containing 0.05% Tween 20) to remove unbound primary antibodies, the membranes were incubated with secondary antibodies at room temperature for 1 h, followed by an additional round of TBST washing. Finally, the target proteins were visualized using enhanced chemiluminescence (ECL) reagents (Biosharp, Beijing, China).

### 2.8. Analysis of the Japanese Encephalitis Virus (JEV) Life Cycle

(1) Adsorption assay

PK-15 cells were seeded in 12-well plates and grown to full confluence. Cells were pre-chilled at 4 °C for 1 h, followed by pretreatment with 2 μM 25HC in a cell culture incubator for 1 h (25HC was not removed). Cells were then inoculated with JEV at an MOI of 0.1 and incubated at 4 °C for 2 h to permit viral adsorption to the cell membrane without internalization. After incubation, cells were washed three times with pre-chilled PBS.

(2) Internalization assay

PK-15 cells were cultured to confluence in 12-well plates and inoculated with JEV at an MOI of 0.1. After incubation at 4 °C for 1 h, unbound virus was removed by washing three times with pre-chilled PBS. Then, 500 μL DMEM supplemented with 2% FBS was added, followed by 2 μM 25HC. Cells were gently mixed and incubated at 4 °C for 2 h to facilitate viral internalization.

Subsequently, the culture medium was discarded, and cells were washed three times with citrate buffer (pH 3.0). Residual acid was neutralized with DMEM after each wash.

(3) Viral replication assay

PK-15 cells were grown to confluence in 12-well plates and inoculated with JEV at an MOI of 0.1. After incubation in a cell culture incubator for 4 h, the medium was removed. Cells were washed three times with citrate buffer (pH 3.0), and residual acid was neutralized with DMEM after each wash.

Then, 500 μL maintenance medium containing 2 μM 25HC was added to each well, and cells were cultured at 37 °C for another 6 h.

(4) Viral release assay

PK-15 cells were cultured to confluence in 12-well plates and inoculated with JEV at an MOI of 0.1. After incubation for 12 h, the culture medium was removed, and cells were washed three times with pre-chilled PBS. Then, 500 μL maintenance medium supplemented with 2 μM 25HC was added, and cells were incubated at 37 °C for a further 2 h.

Samples were harvested for quantification of JEV mRNA levels by qPCR.

### 2.9. Statistical Analysis

Statistical processing and comparative analysis of all experimental datasets were performed using GraphPad Prism 8 software (GraphPad Software, Inc., La Jolla, CA, USA). Normality of data distribution was examined by the Shapiro–Wilk test. All data conformed to a normal distribution and are presented as mean ± SEM. For comparisons between two independent groups, an unpaired Student’s *t*-test was used for statistical analysis. When comparing three or more groups, one-way analysis of variance (ANOVA) was first performed, followed by Tukey’s post hoc test to correct for multiple comparisons and control the false positive rate. All WB experiments were performed with n = 3 independent biological replicates. For each biological replicate, 3 technical replicates were measured by qPCR. A *p*-value of less than 0.05 was defined as statistically significant in this study. Specifically, asterisk symbols were used to mark different levels of statistical significance: * indicated *p* < 0.05, ** indicated *p* < 0.01, and *** indicated *p* < 0.001, respectively. In addition, the abbreviation “ns” was employed to represent a non-significant difference, which corresponds to a *p*-value greater than 0.05.

## 3. Results

### 3.1. 25HC Exerts Antiviral Activity Against JEV at Both Entry and Post-Entry Replication Stages

**Figure 1:**
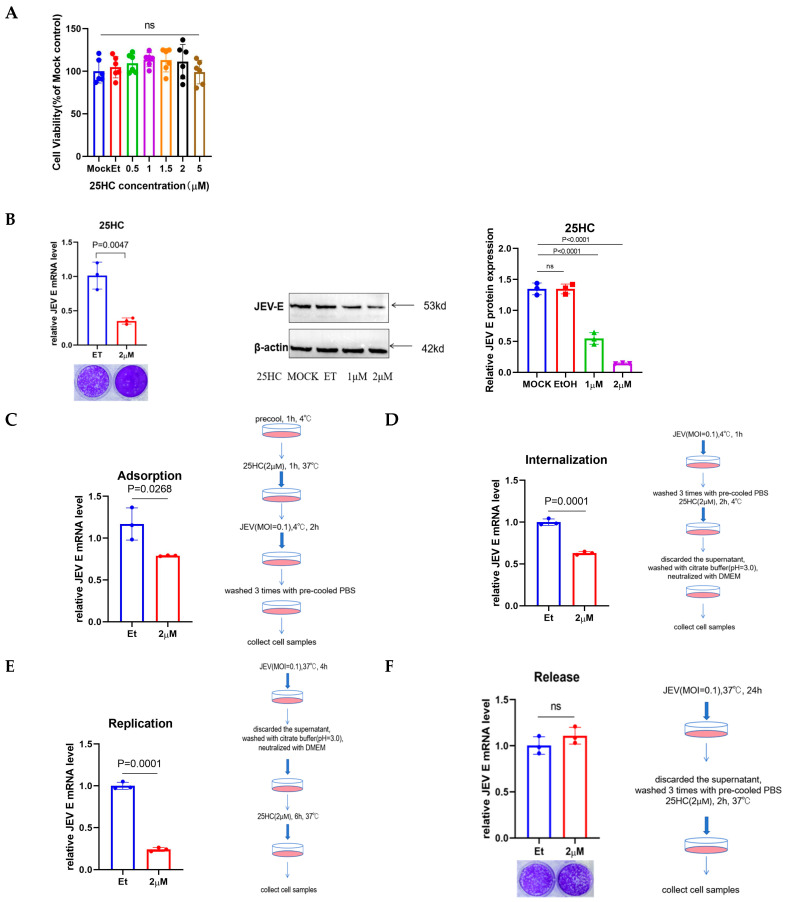
25-Hydroxycholesterol (25HC) inhibits JEV infection at both entry and replication stages. (**A**) Cell viability levels of PK-15 cells exposed to gradually elevated concentrations of 25HC over a 12 h incubation period were assessed using the CCK-8 assay, with the aim of ascertaining non-cytotoxic concentration ranges. (**B**) Antiviral efficacy of 25HC. PK-15 cells were pretreated with vehicle (EtOH) or indicated concentrations of 25HC for 6 h, followed by JEV infection (MOI = 0.1). Viral replication was quantified by qRT-PCR (**left**) and Western blotting (**right**) at 36 h post-infection (hpi). (**C**–**F**) Schematic of the time-of-addition assay demonstrating 25HC inhibition during viral entry and replication phases. PK-15 cells were treated with 25HC (2 μM) at specific stages: adsorption (**C**), internalization (**D**), replication (**E**), and release (**F**). Viral RNA levels were quantified by qRT-PCR. Statistical significance of differences between groups was determined using Student’s *t*-test.

To evaluate the antiviral efficacy of 25-hydroxycholesterol (25HC), we first determined the non-cytotoxic therapeutic window in PK-15 cells. Cytotoxicity assays confirmed that cell viability remained uncompromised at concentrations up to 2 µM (Figure 1A). Within this permissible range, 25HC treatment elicited a potent, dose-dependent suppression of JEV proliferation, as evidenced by a significant reduction in both viral E gene transcription and protein synthesis (Figure 1B). To identify the specific stage of the viral life cycle targeted by 25HC, time-of-addition assays were performed. As illustrated in Figure 1C–F, 25HC treatment effectively restricted viral propagation during both the initial entry phase and the post-entry replication stage. These data indicate that 25HC functions as a broad-phase inhibitor, likely by perturbing the cellular lipid environment essential for multiple steps of JEV infection.

### 3.2. 25HC Imposes a Metabolic Blockade via the Downregulation of the SREBP2-HMGCR Axis

**Figure 2:**
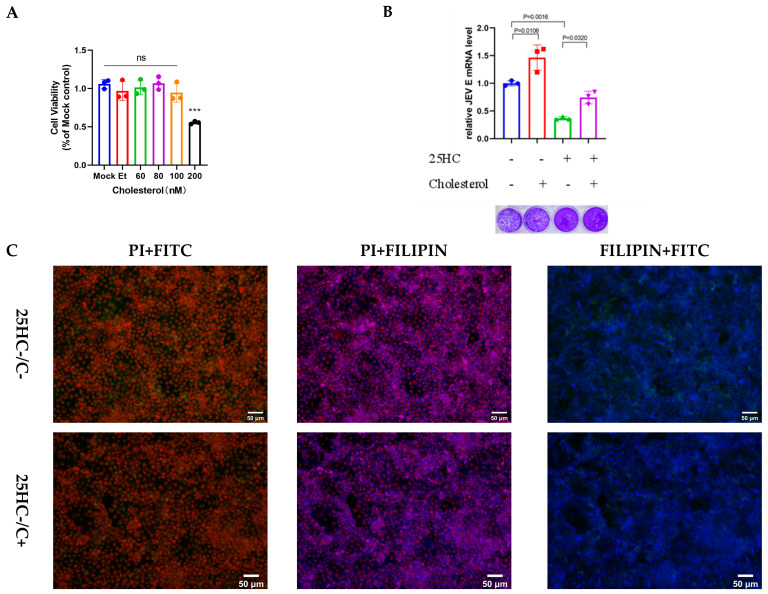

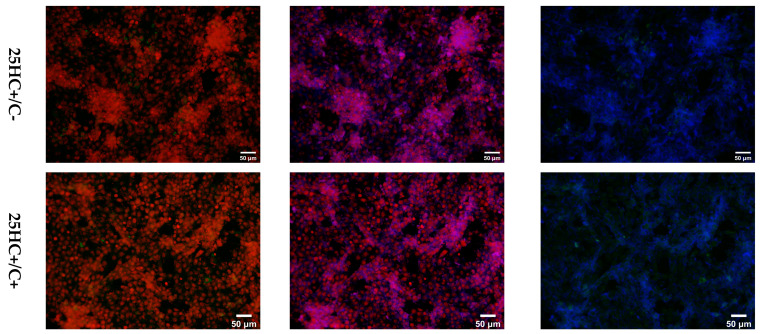
Exogenous cholesterol supplementation rescues JEV infection in 25HC-treated cells. (**A**) Cytotoxicity assessment of exogenous cholesterol on PK-15 cells treated for 12 h (CCK-8 assay). (**B**) Cholesterol rescue assay. PK-15 cells were pretreated with EtOH or 25HC (2 μM) for 6 h, washed, and incubated with specific concentrations of cholesterol for 1 h prior to JEV infection (MOI = 0.1). At 24 hpi, JEV infection levels were assessed by qRT-PCR. (**C**) Representative fluorescence images showing that cholesterol replenishment restores cholesterol distribution (Filipin, blue) and viral antigen expression (green) and nuclei (PI, red) in 25HC-treated cells. Scale bar = 50 μm. All data are presented as mean ± standard deviation (SD) with n = 3. Statistical significance was determined by ANOVA, where *** *p* < 0.001.

25HC thus establishes an antiviral state by cutting off the biosynthetic supply and altering cholesterol distribution. We hypothesized that the antiviral state induced by 25HC is predicated on the depletion of the accessible cholesterol pool. To test this, we performed a cholesterol rescue experiment. Supplementation with exogenous cholesterol (Beyotime, Shanghai, China) successfully reversed the 25HC-mediated reduction in viral RNA and protein load (Figure 2A–C), confirming the critical role of cholesterol availability in sustaining JEV infection.

Mechanistically, we investigated the effect of 25HC on SREBP2, the master regulator of lipid homeostasis. 25HC treatment markedly downregulated the mRNA levels of SREBP2 and its downstream rate-limiting target HMGCR.

**Figure 3:**
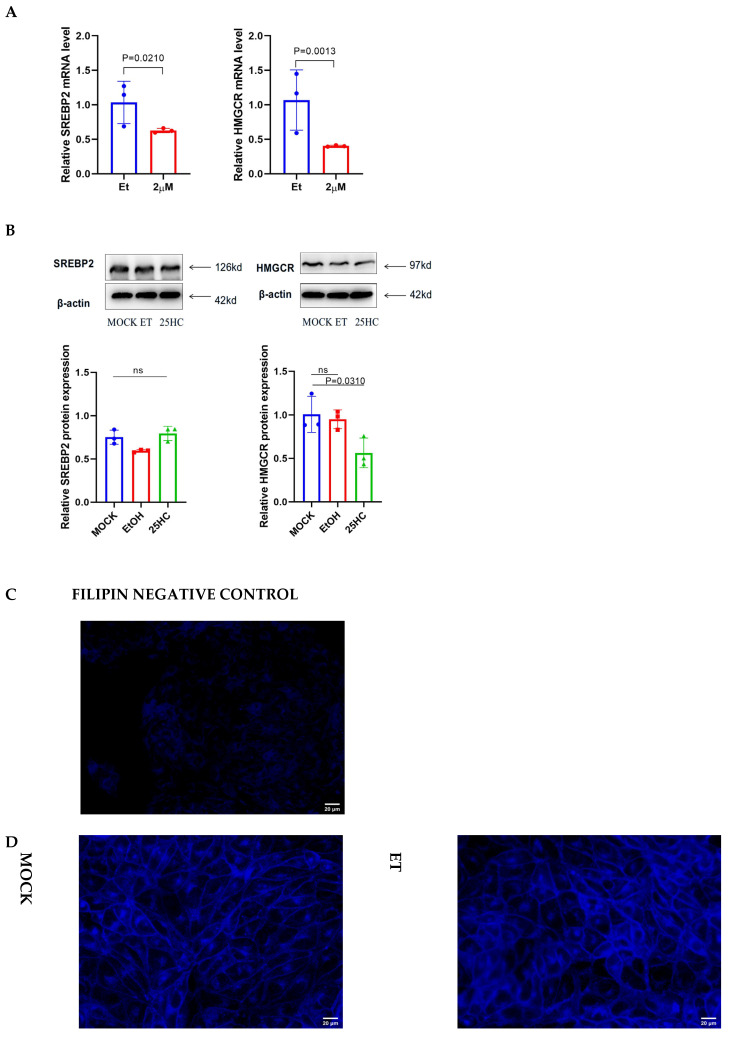

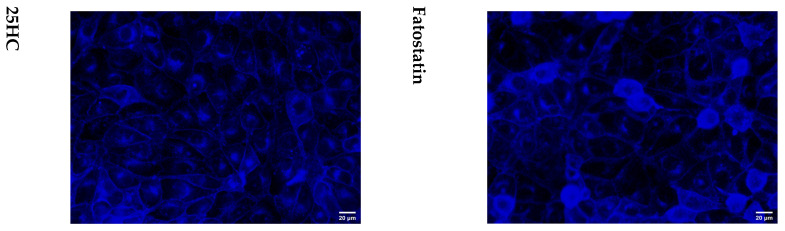
25HC suppresses the expression of the downstream target gene HMGCR via inhibiting SREBP2 activity and alters cholesterol distribution. (**A**,**B**) Expression levels of SREBP2 and HMGCR in PK-15 cells treated with EtOH or indicated concentrations of 25HC for 6 h, measured by qRT-PCR (**A**) and Western blotting (**B**). (**C**) One well was pretreated with 2 mM methyl-β-cyclodextrin (MβCD) for 1 h to remove cholesterol, in order to verify the staining specificity. (**D**) Evaluation of cellular cholesterol distribution. PK-15 cells were treated with 25HC (2 μM) or the SREBP2 inhibitor Fatostatin (2 μM) for 6 h. Cells were fixed and stained with Filipin (blue) to visualize free cholesterol. Representative images show the redistribution of cholesterol from the plasma membrane to the intracellular compartment upon treatment. Scale bar = 20 μm. The bars in the figure represent the mean ± standard deviation (SD) with a sample size of n = 3. Statistical differences were analyzed using Student’s *t*-test.

Interestingly, detection of SREBP2 precursor showed that its protein level remained unchanged, whereas the protein level of downstream HMGCR was decreased. Since the active form of SREBP2 could not be detected, we could only infer that the active form of SREBP2 may be reduced based on the decrease in HMGCR. Furthermore, Filipin staining revealed a topological reorganization of cellular cholesterol: 25HC treatment reduced plasma membrane cholesterol density and sequestered sterols within the cytoplasm (Figure 3C). Collectively, these findings demonstrate that 25HC establishes an antiviral blockade by severing the biosynthetic supply of cholesterol and altering its subcellular distribution.

### 3.3. JEV Infection Hijacks the SREBP2 Pathway to Facilitate Viral Assembly

**Figure 4:**
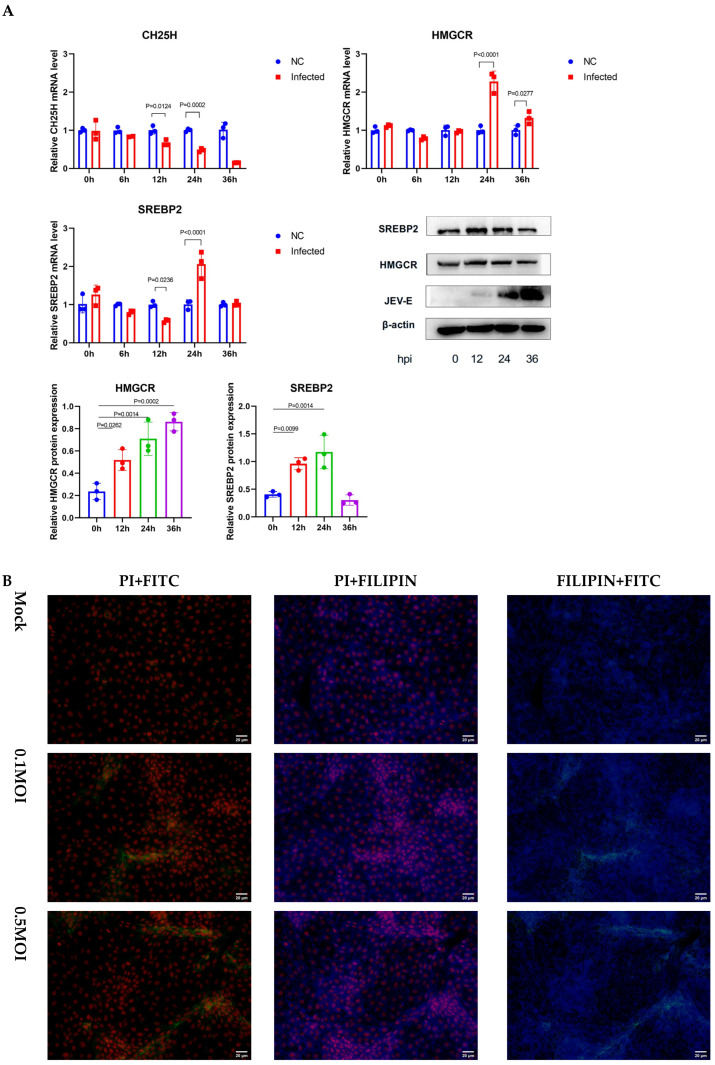
25-Hydroxycholesterol (25HC) downregulates the SREBP2-mediated cholesterol biosynthesis pathway. (**A**) Effect of 25HC on gene expression. PK-15 cells were treated with the indicated concentrations of 25HC for 6 h. The mRNA levels of SREBP2 and its downstream target HMGCR were quantified by qRT-PCR, and protein levels were determined by Western blotting. β-actin was used as a loading control. (**B**) Visualization of intracellular cholesterol distribution. Cells were treated with 25HC (2 μM) for 6 h, fixed, and stained with Filipin (blue) to detect free cholesterol. Scale bar = 20 μm. Note the accumulation of cholesterol in the intracellular compartment and reduction in plasma membrane cholesterol compared to the EtOH control. Data are presented as mean ± SD (n = 3). Statistical significance was analyzed by Student’s *t*-test.

In contrast to the suppressive effects of 25HC, JEV infection drives metabolic reprogramming to favor cholesterol biosynthesis. We observed a time-dependent activation of the SREBP2 signaling axis following viral challenge. The expression levels of both precursor and HMGCR were significantly upregulated within the first 24 h post-infection (hpi) (Figure 4A). To determine whether this metabolic upregulation supports viral structural requirements, we analyzed the spatial relationship between viral components and lipid domains. Fluorescence microscopy analysis demonstrated obvious spatial coincidence between the fluorescent signals of JEV E proteins and cholesterol-enriched microdomains. (Figure 4B). This suggests that the virus actively orchestrates a localized concentration of cholesterol—potentially driven by SREBP2 activation—to facilitate the formation of replication complexes or viral assembly platforms.

### 3.4. Pharmacological Inhibition of SREBP2 Recapitulates the Antiviral State and Abrogates JEV Propagation

**Figure 5:**
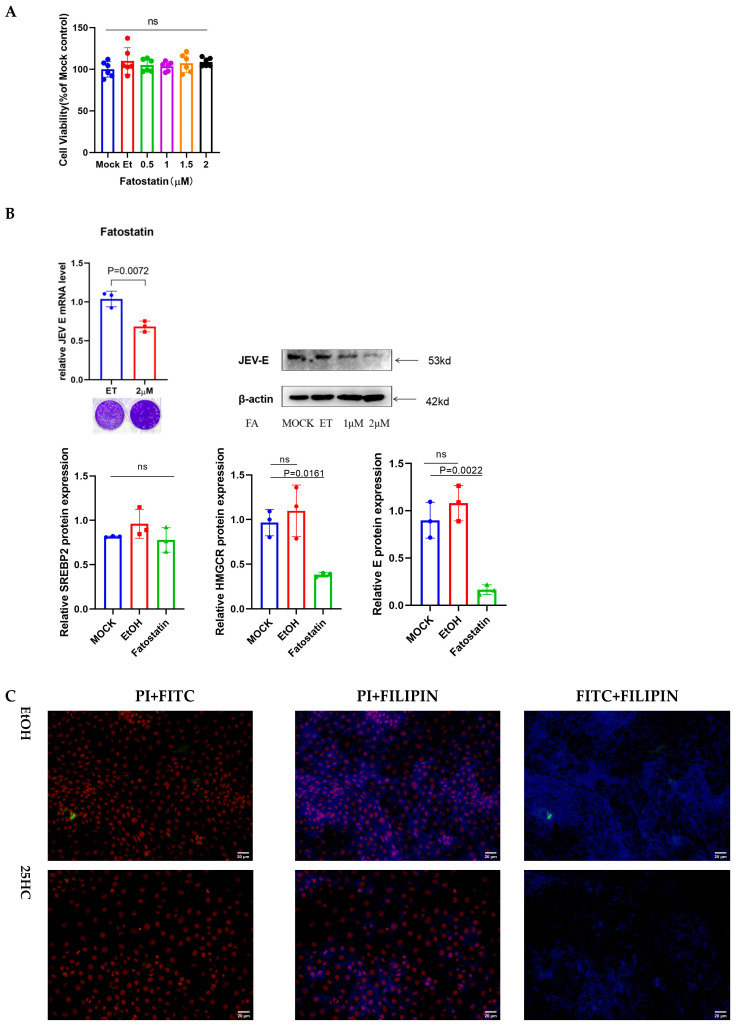

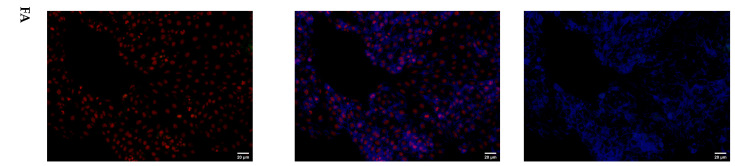
Pharmacological inhibition of SREBP2 by Fatostatin recapitulates the antiviral effect of 25HC. (**A**) Cytotoxicity of Fatostatin on PK-15 cells. Cells were incubated with increasing concentrations of Fatostatin for 12 h, and cell viability was assessed using a CCK-8 assay to determine the maximum non-toxic concentration. (**B**) Antiviral activity of Fatostatin. PK-15 cells were pretreated with Fatostatin (or EtOH vehicle) for 6 h prior to JEV infection (MOI = 0.1). Viral RNA (**left**) and protein (**right**) levels were measured at 36 hpi by qRT-PCR and Western blotting, respectively. (**C**) Comparison of cholesterol distribution. PK-15 cells were treated with either Fatostatin (2 μM) or 25HC (2 μM) for 6 h. Intracellular free cholesterol was visualized by Filipin staining (blue). Representative images show that both Fatostatin and 25HC induce a similar pattern of intracellular cholesterol accumulation. Scale bar = 20 μm. Data are presented as mean ± SD (n = 3). Statistical significance was analyzed by Student’s *t*-test.

To unequivocally validate the SREBP2 pathway as an obligatory dependency for JEV, we utilized Fatostatin [33], a specific inhibitor of SREBP2 translocation. We reasoned that if JEV relies on SREBP2-mediated lipogenesis, chemical blockade of this pathway should mimic the antiviral effects of 25HC. Indeed, Fatostatin treatment dose-dependently abolished viral E protein expression and mRNA accumulation (Figure 5B,C), effectively recapitulating the metabolic restriction observed with 25HC. These results identify the SREBP2-HMGCR axis not merely as a bystander of infection, but as a critical host factor and a viable therapeutic target for metabolic intervention against JEV.

## 4. Discussion

The CH25H-25HC axis functions as a primary innate immune mechanism by restricting cholesterol availability through a dual mode of action. Upon induction, 25HC activates ACAT to esterify accessible cholesterol, thereby reducing the abundance of free cholesterol in the plasma membrane [22]. Concurrently, it suppresses the transcriptional activity of SREBP2 (Figure 3), blocking the compensatory biosynthesis of sterols. This coordinated regulation limits the cellular cholesterol pool, creating a restrictive environment that hampers membrane remodeling processes critical for flavivirus entry and the assembly of replication complexes.

Conversely, JEV infection imposes a substantial demand for intracellular cholesterol to scaffold replication complexes and facilitate virion assembly. Our data reveal that JEV actively counteracts this metabolic blockade. While 25HC treatment downregulates the SREBP2 pathway, JEV infection triggers a significant upregulation of SREBP2 and its downstream targets. This antagonistic regulation aligns with recent findings that JEV actively manipulates host cholesterol metabolism—such as through the inhibition of AMPK signaling—to establish a proviral metabolic state [34]. These findings indicate that the virus must circumvent the host’s metabolic checkpoint to restore the cholesterol biosynthesis necessary for its propagation. JEV infection in mouse embryonic fibroblasts (MEFs) and bone marrow-derived macrophages (BMDMs) results in a significant transcriptional downregulation of genes involved in the cholesterol biosynthetic pathway, including HMGCR, DHCR7, and SREBP2 [35]. Similarly, JEV infection downregulates SREBP-2 mRNA in HEK293T cells, which is consistent with the downmodulation of its intronic microRNA, miR-33a-5p [36]. Although the expression of the cholesterol biosynthetic pathway varies during JEV infection, viral infection is a dynamic process, and subtle changes in intracellular cholesterol levels can affect viral infection. JEV exerts a highly precise regulatory effect on intracellular cholesterol. Notably, our observations of cholesterol subcellular redistribution and its spatial overlap with JEV E proteins were obtained using wide-field fluorescence microscopy combined with filipin staining for free cholesterol. This technical approach has inherent limitations in spatial resolution and signal contrast compared with confocal laser scanning microscopy, which precludes high-precision quantitative colocalization analysis (e.g., calculation of colocalization coefficients) between viral proteins and cholesterol-enriched microdomains. Thus, all descriptions of cholesterol spatial distribution and its association with JEV E proteins in this study are presented as qualitative morphological evidence rather than quantitative data, with no quantitative inferences made regarding the degree of their spatial correlation. Currently, numerous novel approaches are available to investigate how JEV infects cells under dynamic conditions, suggesting that there is great potential for further research on the interaction between JEV and cholesterol metabolism.

25HC serves as an important regulatory factor in cholesterol metabolism and plays a mediating role in cholesterol trafficking from the plasma membrane to the endoplasmic reticulum [37]. While our results confirm this effect, the time-of-addition assays demonstrate that 25HC also exerts inhibition during the post-entry replication stage. By comparing the gene expression profiles—where 25HC suppresses but JEV induces the cholesterol biosynthetic pathway—our data suggest that the SREBP2-HMGCR axis may act as a critical regulatory hub in this crosstalk under our in vitro experimental conditions. The capacity of 25HC to inhibit JEV replication appears largely attributable to its ability to block SREBP2 activation, which is required for optimal JEV replication.

The dependency of JEV on this pathway was definitively confirmed through pharmacological intervention. Treatment with Fatostatin, a specific inhibitor of SREBP2 translocation, recapitulated the antiviral phenotype observed with 25HC and effectively abolished viral propagation. This validates the SREBP2-HMGCR axis as a viable target for host-directed therapy. Furthermore, the therapeutic potential extends to neuroprotection. Recent evidence suggests that inflammation-induced activation of SREBP2 contributes to blood–brain barrier (BBB) dysfunction, whereas 25HC treatment can attenuate this breakdown by modulating the SREBP2 pathway [38]. Therefore, targeting SREBP2 represents a feasible “dual-hit” strategy: it restricts neurotropic flaviviruses by limiting their essential cholesterol supply while simultaneously reinforcing CNS integrity against inflammation-mediated damage. This study was conducted only in PK-15 cells; further in vivo experiments in animal models are required to validate the therapeutic potential of 25HC against JEV.

## Figures and Tables

**Table 1:** Primer sequences for qRT-PCR.

Target Names	(5′ → 3′) Primer Sequences
JEV-E-F	CAGTGGAGCCACTTGGGTG
JEV-E-R	TTGTGAGCTTCTCCTGTCG
SREBF2-F	TGGAGCAGCCTCAATGTCAG
SREBF2-R	TTCGTGCAGAAACACCTTGC
CH25H-F	CACTCACAGACTAGTACCTTTCG
CH25H-R	TCCCAGTATTTTGTCCCAGTG
HMGCR-F	AGCTCCAACTCACAGGATGAA
HMGCR-R	ACGAAGTAGGTGGCGAGAAC
β-actin-F	CTTCCTGGGCATGGAGTCC
β-actin-R	GGCGCGATGATCTTGATCTTC

## Data Availability

The original contributions presented in this study are included in the article. Further inquiries can be directed to the corresponding author.

## References

[1] Solomon T., Ni H., Beasley D.W.C., Ekkelenkamp M., Cardosa M.J., Barrett A.D.T. (2003). Origin and Evolution of Japanese Encephalitis Virus in Southeast Asia. J. Virol..

[2] Wang Y., Li Y., Ding T. (2017). Heat Shock Protein 90β in the Vero Cell Membrane Binds Japanese Encephalitis Virus. Int. J. Mol. Med..

[3] Samy A.M., Alkishe A.A., Thomas S.M., Wang L., Zhang W. (2018). Mapping the Potential Distributions of Etiological Agent, Vectors, and Reservoirs of Japanese Encephalitis in Asia and Australia. Acta Trop..

[4] Mohsin F., Suleman S., Anzar N., Narang J., Wadhwa S. (2022). A Review on Japanese Encephalitis Virus Emergence, Pathogenesis and Detection: From Conventional Diagnostics to Emerging Rapid Detection Techniques. Int. J. Biol. Macromol..

[5] Wang X., Li S.-H., Zhu L., Nian Q.-G., Yuan S., Gao Q., Hu Z., Ye Q., Li X.-F., Xie D.-Y. (2017). Near-Atomic Structure of Japanese Encephalitis Virus Reveals Critical Determinants of Virulence and Stability. Nat. Commun..

[6] Reyes-Del Valle J., Chávez-Salinas S., Medina F., Del Angel R.M. (2005). Heat Shock Protein 90 and Heat Shock Protein 70 Are Components of Dengue Virus Receptor Complex in Human Cells. J. Virol..

[7] Lee C.-J., Liao C.-L., Lin Y.-L. (2005). Flavivirus Activates Phosphatidylinositol 3-Kinase Signaling to Block Caspase-Dependent Apoptotic Cell Death at the Early Stage of Virus Infection. J. Virol..

[8] Soto-Acosta R., Bautista-Carbajal P., Cervantes-Salazar M., Angel-Ambrocio A.H., Del Angel R.M. (2017). DENV Up-Regulates the HMG-CoA Reductase Activity through the Impairment of AMPK Phosphorylation: A Potential Antiviral Target. PLoS Pathog..

[9] Bautista-Olivier C.D., Murillo-González F.E., Limón-Pacheco J., Hernández-Cázares F., Palacios-Rápalo S.N., Cordero-Rivera C.D., Farfan-Morales C.N., del Ángel R.M., Elizondo G. (2025). The Pregnane X Receptor Is a Novel Host Target for Dengue Virus Infection That Reprograms Lipid Metabolism and Suppresses the Immune Response. Biochem. Pharmacol..

[10] Mackenzie J.M., Khromykh A.A., Parton R.G. (2007). Cholesterol Manipulation by West Nile Virus Perturbs the Cellular Immune Response. Cell Host Microbe.

[11] Yun S.-I., Lee Y.-M. (2018). Early Events in Japanese Encephalitis Virus Infection: Viral Entry. Pathogens.

[12] Das S., Chakraborty S., Basu A. (2010). Critical Role of Lipid Rafts in Virus Entry and Activation of Phosphoinositide 3′ Kinase/Akt Signaling during Early Stages of Japanese Encephalitis Virus Infection in Neural Stem/Progenitor Cells. J. Neurochem..

[13] Heaton N.S., Randall G. (2011). Multifaceted Roles for Lipids in Viral Infection. Trends Microbiol..

[14] Bauman D.R., Bitmansour A.D., McDonald J.G., Thompson B.M., Liang G., Russell D.W. (2009). 25-Hydroxycholesterol Secreted by Macrophages in Response to Toll-like Receptor Activation Suppresses Immunoglobulin A Production. Proc. Natl. Acad. Sci. USA.

[15] Liu S.-Y., Aliyari R., Chikere K., Li G., Marsden M.D., Smith J.K., Pernet O., Guo H., Nusbaum R., Zack J.A. (2013). Interferon-Inducible Cholesterol-25-Hydroxylase Broadly Inhibits Viral Entry by Production of 25-Hydroxycholesterol. Immunity.

[16] Russell D.W. (2000). Oxysterol Biosynthetic Enzymes. Biochim. Biophys. Acta.

[17] Raniga K., Liang C. (2018). Interferons: Reprogramming the Metabolic Network against Viral Infection. Viruses.

[18] Archana K., Qazi B., Bohra B., Tripathi R.K., Haldar S. (2025). 25-Hydroxy Cholesterol Effectively Inhibits Japanese Encephalitis Virus Infection in a Cellular Model. Biochem. Biophys. Res. Commun..

[19] Bielska A.A., Olsen B.N., Gale S.E., Mydock-McGrane L., Krishnan K., Baker N.A., Schlesinger P.H., Covey D.F., Ory D.S. (2014). Side-Chain Oxysterols Modulate Cholesterol Accessibility through Membrane Remodeling. Biochemistry.

[20] Kandutsch A.A., Chen H.W. (1975). Regulation of Sterol Synthesis in Cultured Cells by Oxygenated Derivatives of Cholesterol. J. Cell. Physiol..

[21] Branche E., Wang Y.-T., Viramontes K.M., Valls Cuevas J.M., Xie J., Ana-Sosa-Batiz F., Shafee N., Duttke S.H., McMillan R.E., Clark A.E. (2022). SREBP2-Dependent Lipid Gene Transcription Enhances the Infection of Human Dendritic Cells by Zika Virus. Nat. Commun..

[22] Heisler D.B., Johnson K.A., Ma D.H., Ohlson M.B., Zhang L., Tran M., Corley C.D., Abrams M.E., McDonald J.G., Schoggins J.W. (2023). A Concerted Mechanism Involving ACAT and SREBPs by Which Oxysterols Deplete Accessible Cholesterol to Restrict Microbial Infection. eLife.

[23] Visoso-Carvajal G., García-Cordero J., Ybalmea-Gómez Y., Diaz-Flores M., León-Juárez M., Hernández-Rivas R., Nava P., Villegas-Sepúlveda N., Cedillo-Barrón L. (2025). Interplay Between NLRP3 Activation by DENV-2 and Autophagy and Its Impact on Lipid Metabolism in HMEC-1 Cells. Pathogens.

[24] Yang S., He M., Liu X., Li X., Fan B., Zhao S. (2013). Japanese Encephalitis Virus Infects Porcine Kidney Epithelial PK15 Cells via Clathrin- and Cholesterol-Dependent Endocytosis. Virol. J..

[25] Niu J., Jiang Y., Xu H., Zhao C., Zhou G., Chen P., Cao R. (2018). TIM-1 Promotes Japanese Encephalitis Virus Entry and Infection. Viruses.

[26] Chandan K., Gupta M., Sarwat M. (2019). Role of Host and Pathogen-Derived MicroRNAs in Immune Regulation During Infectious and Inflammatory Diseases. Front. Immunol..

[27] Xu W., Yang K., Zheng Y., Cao S., Yan Q., Huang X., Wen Y., Zhao Q., Du S., Lang Y. (2023). BAK-Mediated Pyroptosis Promotes Japanese Encephalitis Virus Proliferation in Porcine Kidney 15 Cells. Viruses.

[28] Zhou X., Yuan Q., Zhang C., Dai Z., Du C., Wang H., Li X., Yang S., Zhao A. (2021). Inhibition of Japanese Encephalitis Virus Proliferation by Long Non-Coding RNA SUSAJ1 in PK-15 Cells. Virol. J..

[29] Zhao C., Liu H., Xiao T., Wang Z., Nie X., Li X., Qian P., Qin L., Han X., Zhang J. (2020). CRISPR Screening of Porcine sgRNA Library Identifies Host Factors Associated with Japanese Encephalitis Virus Replication. Nat. Commun..

[30] Mukhopadhyay S., Kuhn R.J., Rossmann M.G. (2005). A Structural Perspective of the Flavivirus Life Cycle. Nat. Rev. Microbiol..

[31] Nain M., Abdin M.Z., Kalia M., Vrati S. (2016). Japanese Encephalitis Virus Invasion of Cell: Allies and Alleys. Rev. Med. Virol..

[32] Luo S.-Q., Cao S.-J., Zhao Q. (2024). CRISPR/Cas9-Mediated Knockout of the HuR Gene in U251 Cell Inhibits Japanese Encephalitis Virus Replication. Microorganisms.

[33] Li M., Lu Q., Zhu Y., Fan X., Zhao W., Zhang L., Jiang Z., Yu Q. (2022). Fatostatin Inhibits SREBP2-Mediated Cholesterol Uptake via LDLR against Selective Estrogen Receptor α Modulator-Induced Hepatic Lipid Accumulation. Chem. Biol. Interact..

[34] Zhou J., Zhang M., Wang Q., Li M., Bai J., Dai Q., Zhang Y., Yan M., Li X., Chen J. (2024). Two Novel Compounds Inhibit Flavivirus Infection in Vitro and in Vivo by Targeting Lipid Metabolism. J. Virol..

[35] Khera S., Sharma K.B., Kumar Y., Kalia M. (2026). Downmodulation of Cholesterol Biosynthetic Network Governs Activation of the Innate Immune Response to Japanese Encephalitis Virus Infection. J. Virol..

[36] Chen Z., Ye J., Ashraf U., Li Y., Wei S., Wan S., Zohaib A., Song Y., Chen H., Cao S. (2016). MicroRNA-33a-5p Modulates Japanese Encephalitis Virus Replication by Targeting Eukaryotic Translation Elongation Factor 1A1. J. Virol..

[37] Zhao J., Chen J., Li M., Chen M., Sun C. (2020). Multifaceted Functions of CH25H and 25HC to Modulate the Lipid Metabolism, Immune Responses, and Broadly Antiviral Activities. Viruses.

[38] Loiola R.A., Nguyen C., Dib S., Saint-Pol J., Dehouck L., Sevin E., Naudot M., Landry C., Pahnke J., Pot C. (2024). 25-Hydroxycholesterol Attenuates Tumor Necrosis Factor Alpha-Induced Blood-Brain Barrier Breakdown in Vitro. Biochim. Biophys. Acta BBA-Mol. Basis Dis..

